# Supplementary material for: Splice-Junction-Based Mapping of Alternative Isoforms in the Human Proteome
Source: Cell Rep. Author manuscript; Available in PMC 2020 Jan 15. (PMC6961840; doi:10.1016/j.celrep.2019.11.026)
Supplement: 2 [file NIHMS1546469-supplement-2.zip › DF1/PXD010154/Spleen/Spleen_16_RNF123_GANTSTTSSAEDAGHLLAAARLPADHFSRK.pdf]

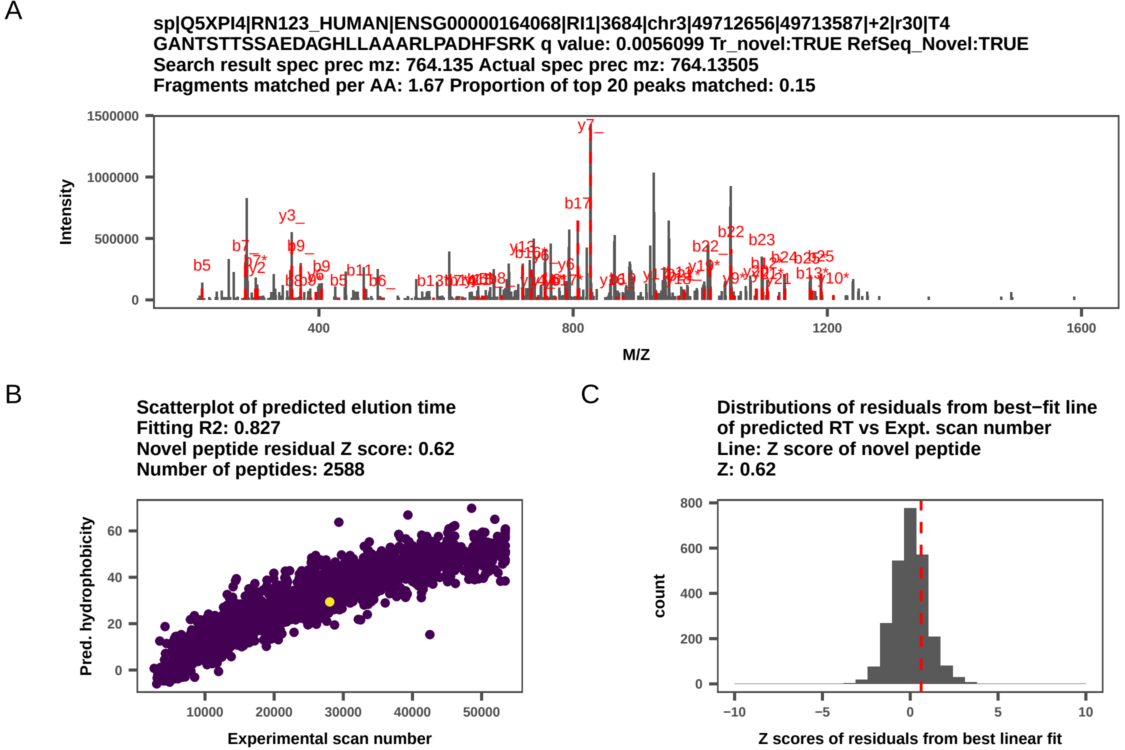
A
sp|Q5XPI4|RN123_HUMAN|ENSG00000164068|RI1|3684|chr3|49712656|49713587|+2|r30|T4
GANTSTTSSAEDAGHLLAAARLPADHFSRK q value: 0.0056099 Tr_novel:TRUE RefSeq_Novel:TRUE
Search result spec prec mz: 764.135 Actual spec prec mz: 764.13505
Fragments matched per AA: 1.67 Proportion of top 20 peaks matched: 0.15
Intensity
1500000
1000000
500000
0
M/Z
400
800
1200
1600
y7_
b17
y3_
b22
b23
b22_
b5
b7_
b9_
b11
b13*
y10*
b24
B
Scatterplot of predicted elution time
Fitting R2: 0.827
Novel peptide residual Z score: 0.62
Number of peptides: 2588
Pred. hydrophobicity
60
40
20
0
10000
20000
30000
40000
50000
Experimental scan number
C
Distributions of residuals from best−fit line
of predicted RT vs Expt. scan number
Line: Z score of novel peptide
Z: 0.62
count
800
600
400
200
0
−10
−5
0
5
10
Z scores of residuals from best linear fit
